# BV-2 Microglial Cells Respond to Rotenone Toxic Insult by Modifying Pregnenolone, 5α-Dihydroprogesterone and Pregnanolone Levels

**DOI:** 10.3390/cells9092091

**Published:** 2020-09-13

**Authors:** Rossella Avallone, Chiara Lucchi, Giulia Puja, Alessandro Codeluppi, Monica Filaferro, Giovanni Vitale, Cecilia Rustichelli, Giuseppe Biagini

**Affiliations:** 1 Department of Life Sciences, Modena and Reggio Emilia University, 41125 Modena, Italy; giulia.puja@unimore.it (G.P.); alessandrocodeluppi@unimore.it (A.C.); giovanni.vitale@unimore.it (G.V.); cecilia.rustichelli@unimore.it (C.R.); 2 Department of Biomedical, Metabolic and Neural Sciences, University of Modena and Reggio Emilia, 41125 Modena, Italy; lucchi.chiara86@gmail.com (C.L.); monica.filaferro@unimore.it (M.F.); giuseppe.biagini@unimore.it (G.B.)

**Keywords:** steroidogenesis, microglia, BV-2 cells, neurosteroids, rotenone, ROS, neuroinflammation, neurodegeneration

## Abstract

Neuroinflammation, whose distinctive sign is the activation of microglia, is supposed to play a key role in the development and progression of neurodegenerative diseases. The aim of this investigation was to determine levels of neurosteroids produced by resting and injured BV-2 microglial cells. BV-2 cells were exposed to increasing concentrations of rotenone to progressively reduce their viability by increasing reactive oxygen species (ROS) production. BV-2 cell viability was significantly reduced 24, 48 and 72 h after rotenone (50–1000 nM) exposure. Concomitantly, rotenone (50–100 nM) determined a dose-independent augmentation of ROS production. Then, BV-2 cells were exposed to a single, threshold dose of rotenone (75 nM) to evaluate the overtime release of neurosteroids. In particular, pregnenolone, pregnenolone sulfate, progesterone, 5α-dihydroprogesterone (5α-DHP), allopregnanolone, and pregnanolone, were quantified in the culture medium by liquid chromatography with tandem mass spectrometry (LC-MS/MS) analysis. BV-2 cells synthesized all the investigated neurosteroids and, after exposure to rotenone, 5αDHP and pregnanolone production was remarkably increased. In conclusion, we found that BV-2 cells not only synthesize several neurosteroids, but further increase this production following oxidative damage. Pregnanolone and 5α-DHP may play a role in modifying the progression of neuroinflammation in neurodegenerative diseases.

## 1. Introduction 

Microglia are natural immune cells composing up to 10% of the central nervous system (CNS) population, and up to 20% of glial cells [1,2,3]. Microglia have been shown to be implicated in development and maintenance of CNS homeostasis, but they become activated and contribute to neuroinflammation and neurodegeneration in case of injury to the nervous tissue [4,5,6]. Specifically, in response to injury microglial cells develop a variety of reactive phenotypes to tentatively reestablish brain homeostasis and confine neuronal damage [7,8]. In addition to neuronal damage, microglia reactivity can be induced by exposure to neurotoxic agents in a manner independent of neuronal lesion [1]. Indeed, microglia are dynamic cells that, when activated, change not only morphology, but also receptor expression and secretion of chemokines and cytokines. In vivo, many different types of microglia phenotypes could be identified [9,10,11]. However, in vitro microglial activation produces the differentiation into a pro-inflammatory phenotype (M1) or an anti-inflammatory phenotype (M2). The M1/M2 polarization was originally established in peripheral macrophages and then adapted to primary cultured microglia because they behave like peripheral monocytes by losing their characteristic microglial gene signature. After the development of cell type-specific isolation techniques for microglia, next generation sequencing and, more recently, single cell RNA sequencing, it was clear that the classic M1/M2 polarization was not able to recapitulate the actual microglial phenotype in the brain. Indeed, when characterized in vivo, microglia span from a homeostatic to a disease-associated/neurodegenerative phenotype [12]. However, in the present study, we considered the classical M1/M2 polarization paradigm, which is still applicable for in vitro studies even though not directly relevant to the in vivo behavior of microglia. Phenotype M1 produces pro-inflammatory cytokines such as interleukin (IL)-1β and tumor necrosis factor (TNF)-α and expresses nicotinamide-adenine dinucleotide phosphate (NADPH) oxidase, which generates superoxide and reactive oxygen species (ROS) [5]. On the other hand, the M2 phenotype releases anti-inflammatory factors such as IL-10 and transforming growth factor (TGF)-β, as well as other growth factors and neurotrophic factors [5]. In neurological diseases such as Parkinson’s disease (PD) [13], Alzheimer’s disease (AD) [14,15] and multiple sclerosis (MS) [16], microglia are rapidly activated to acquire a M1 phenotype [17]. Notably, these neurodegenerative diseases share as a common feature the reduction in levels of the neurosteroid allopregnanolone [18,19,20].

Neurosteroidogenesis starts with the transfer of cholesterol into the mitochondria, an initial step involving translocator protein (TSPO) and the steroidogenic acute regulatory protein (StAR) [21]. This is then followed by conversion of cholesterol to pregnenolone by the mitochondrial cleavage enzyme of the side chain, cytochrome P450scc. Pregnenolone is released by mitochondria and, when present in excess, is metabolized to pregnenolone sulfate by the steroid sulfotransferase. More properly, pregnenolone is oxidized to progesterone by the 3β-hydroxysteroid dehydrogenase enzyme (3β-HSD). Subsequently, the enzyme 5α-reductase converts progesterone to 5α-dihydroprogesterone (5α-DHP), which in turn is reduced to allopregnanolone by 3α-HSD. Alternatively, progesterone is converted by 5β-reductase to 5β-dihydroprogesterone (5β-DHP), which in turn is reduced to obtain pregnanolone by the enzyme 3β-HSD. Due to their lipophilic structure, neurosteroids can be transferred from one cell to another and, consequently, their metabolism is further changed by the various types of enzymes expressed in the other cell types [22], including both neurons and glia [23,24].

Even if classically found in the adrenal glands and gonads, most steroidogenic enzymes are also present in the CNS, where their expression is specific for cell type, region, stage of development, and gender [25]. 3β-HSD, in fact, is expressed in cultured human oligodendroglial, astroglial, and neuronal cell lines, indicating that several types of cells have the capacity of synthesizing progesterone [26].

Neurosteroid synthesis can be markedly affected by neuroinflammation, which in turn can be regulated by several neurosteroids [27]. In particular, 5α-reductase has been found to be expressed in microglia [22], but only pregnenolone was found to be produced by cultivated microglia BV-2 cells [28]. Indeed, glial cells, such as oligodendrocytes and astrocytes, were initially identified as the main site for the biosynthesis of neurosteroids in the brain of mammals and other vertebrates [25]; however, no investigation so far demonstrated the full availability of neurosteroid synthesis in microglia, especially of allopregnanolone and pregnanolone which are well-known modulators of neurotransmission [28].

Therefore, we investigated the synthesis of neurosteroids in microglial BV-2 cells in basal conditions and after exposure to the neurotoxin rotenone, which induces the production of free radicals by mitochondria with consequent oxidative damage and ROS production. We used mouse BV-2 cells, which have been generated by infecting primary microglial cell cultures with a v-*raf*/v-*myc* oncogene carrying retrovirus (J2) [29] and are recognized to express TSPO and have steroidogenic ability [30]. BV-2 cells are an effective substitute for primary cultures. When exposed to lipopolysaccharides, 90% of the genes induced by primary microglia were also induced by BV-2 cells; nevertheless, the upregulation of genes in BV-2 was less marked than in primary microglia [31]. Additionally, BV-2 microglial cells are used as an in vitro model of neuroinflammation [32,33,34], so they can be suitable to study neurodegenerative disorders such as AD and PD.

## 2. Materials and Methods

### 2.1. Chemicals and Reagents

RPMI 1640 with and without phenol red, glutamine, penicillin, streptomycin, fetal bovine serum, 3-(4,5-dimethylthiazol-2-yl)-2,5-diphenyltetrazolium bromide (MTT) and rotenone were purchased from Merck Life Science (Milan, Italy); fluorogenic probes CellROX^®^ Green Reagent was purchased from Life Technologies, Milan, Italy. All other chemical reagents were HPLC grade.

Certified standard for the target neurosteroids and isotope-labeled internal standards (allopregnanolone-2,2,3,4,4-d5 and pregnenolone-20,21-13C2-16,16-d2 sulfate sodium salt) were supplied as pure substances or solutions (100 µg/mL) from Merck Life Science (Milan, Italy). AmplifexTM-Keto Reagent Kit and Discovery DSC-18 SPE cartridges (100 mg; 1 mL) were also purchased from Merck Life Science (Milan, Italy). Acetonitrile (ACN), methanol, formic acid (FA) and ammonium formate (AmmF) were of liquid chromatography-mass spectrometry (LC-MS) purity grade (Merck Life Science, Milan, Italy); ultra-pure water was obtained by a Milli-Q Plus185 system (Millipore, Milford, MA, USA).

### 2.2. Cell Culture

Murine BV-2 microglia cells were kindly provided by Prof. Elisabetta Blasi (University of Modena and Reggio Emilia, Italy). The cells were cultured in RPMI 1640 containing glutamine (2 mM), penicillin (100 U/mL), streptomicyn (100 µg/mL) supplemented with 10% fetal bovine serum (FBS). BV-2 cells were incubated in a humidified atmosphere containing 5% CO2 at 37 °C. All subsequent treatments were performed using serum-free media. For all experiments, cells were grown to 80–90% confluency and then subjected to no more than 20 cell passages.

### 2.3. Viability of BV-2 Microglial Cells Exposed to Rotenone

To evaluate the cell viability of BV-2 exposed to rotenone (1–1000 nM) MTT assay was performed with modifications [35]. 10,000 BV-2 cells were seeded in 96 well-plates. After 24 h the cells were exposed with increasing concentrations of rotenone in serum-free medium, without phenol red and incubated for 24, 48 and 72 h. Afterwards, 3-(4,5-dimethylthiazol-2-yl)-2,5-diphenyltetrazolium bromide (MTT) solution was added to each well and incubated for 2 h. Finally, 10% sodium dodecyl sulfate solution was added to dissolve formazan and absorbance was measured at 570 nm, using 620 nm as wavelength reference.

### 2.4. Viability Evaluation of BV-2 Cells for Neurosteroids Dosage Normalization

The trypan blue exclusion dye assay was performed according to the manufacturer’s instructions (Merck Life Science, Milan, Italy). Briefly, cells were scraped off and were re-suspended in fresh medium and 100 µL sample was collected for cell counting. Cells were stained with trypan blue at a final concentration of 0.05%. The numbers of dead (blue) and living (white) cells were scored under light microscopy in a Bürker chamber. This assay was performed for cell counting in order evaluate, for each sample, the number of living cells in comparison to the total number of cells for neurosteroids quantification.

### 2.5. Determination of ROS

The ROS generation was determined using the fluorogenic probes CellROX^®^ Green Reagent according to the manufacturer’s instructions. Briefly BV-2 cells were seeded in a 96-well plate (at a density of 10,000 cells/well) and maintained in complete culture media for 24 h. Then, they were pretreated for 4 h with rotenone (50–100 nM). Successively, 10 µL of CellROX^®^ Green Reagent were added in each well 30 min before the end of treatment at the concentration of 5 µM. The emitted fluorescence intensity was measured using a fluorskan Ascent FL (Biorad; Milan, Italy) with wavelengths of 485 nm (excitation) and 520 nm (emission).

### 2.6. Sample Processing

For the neurosteroids determinations, 100,000 BV-2 cells/well were seeded in 24 well plate in serum free RPMI 1640 without phenol red. After 24 h they were exposed to the 75 nM rotenone for 24, 48, 72 h. At the end of the treatment the medium was aspirated and centrifuged to eliminate the cells in suspension and the supernatant was used for the neurosteroids determination.

Aliquots (700 µL) of medium samples were spiked with 50 µL of the internal standard solution, vortexed (90 s) and purified by C-18 SPE procedure to remove inorganic salts present in the cell medium, which may suppress the signal for the target analytes during LC-MS/MS analyses.

Briefly, the samples were loaded on the C-18 cartridges previously conditioned with methanol (1.0 mL) and water (1.0 mL); the inorganic salts were removed with a water wash (2 × 500 µL) and the neurosteroids were finally eluted with methanol (2 × 500 µL). The obtained eluates were evaporated to dryness by a Mod. The 5305 Concentrator Plus (Eppendorf AG, Hamburg, Germany) and subjected to derivatization with Amplifex Keto Reagent (50 µL) for one hour at room temperature and in the dark. The obtained samples were then added with 50 µL methanol/water (70/30), centrifuged (21.300× *g*, 10 min, 10 °C) and subjected to LC-MS/MS analysis.

### 2.7. Working Solutions and Calibrators

A stock solution containing all the target neurosteroids was serially diluted with methanol to obtain working solutions at ten concentration levels. A stock solution of the isotope-labeled internal standards (ISs) was prepared in methanol at a concentration of 1000 pg/mL for both the ISs. All solutions were stored at −20 °C until use.

Aliquots (700 µL) of blank cell medium were spiked with 50 µL of the internal standards solution and 50 µL of the working solutions to obtain calibration samples (*n* = 10) in the range 5.0 ± 1250 pg/700 µL for pregnenolone and 5α-DHP, 1.0 ± 250 pg/700 µL for pregnanolone and 0.2 ± 50 pg/700 µL for pregnenolone sulfate, progesterone, and allopregnanolone. The obtained calibrators were processed as described above and analyzed in triplicate on three separate days.

### 2.8. LC-MS/MS Conditions

Analyses were carried out on an Agilent 1200 Series HPLC system comprised of a binary pump, vacuum degasser, thermostated autosampler and column oven (Agilent, Waldbronn, Germany). The analytical column was a Kinetex XB-C18 column (100 × 2.1 mm; 2.6 µm particle size) equipped with a UHPLC C18 SecurityGuard cartridge (2.1 mm) (Phenomenex, Torrance, CA, USA).

The mobile phase consisted of (A) water/ACN 90/10 (*v*/*v*; + 3 mM AmmF, + 0.1% FA) and (B) ACN/water 90/10 (*v*/*v*; + 3 mM AmmF, 0.1% FA); the optimized elution program was as follows: 1.5 min isocratic step at 20% (B), linear increase from 20 to 25% (B) in 0.5 min, linear increase from 25 to 45% (B) in 9 min, 3.0 min isocratic step at 45% (B), linear gradient from 45 to 90% (B) in 1 min, 10.0 min isocratic step at 90% (B), linear gradient from 90 to 20% (B) in 1 min. The column was re-equilibrated at 20% mobile phase B for 10 min before the next injection. The flow-rate was 0.3 mL/min and a pre-equilibration period of 10 min was used between each run. The injection volume was 10 µL; the column temperature was set at 40°C and the autosampler was maintained at 4 °C.

Mass spectrometric detection was conducted using an Agilent QQQ-MS/MS (6410B) triple quadrupole mass analyzer operating in ESI positive ion mode under multiple reaction monitoring conditions (MRM). The ion source parameters were the following: capillary voltage, 3000 V; nebulizer (N_2_) pressure, 30 psi; drying gas (N_2_) temperature, 350 °C; drying gas flow, 10 L/min; collision gas (N_2_) pressure, 15 psi; electron multiplier voltage, 700 eV; dwell time, 30 msec. Instrument control, data acquisition, qualitative and quantitative data analysis were performed by MassHunter workstation software, version B.05.00 (Agilent, Waldbronn, Germany). The MS/MS acquisition parameters were optimized by direct infusion of each derivatized analyte and IS (50,000 pg/mL; flow: 10 µL/min).

Three MRM transitions were monitored for each compound; the most intense transition or the transition with the lower level of background noise was selected for quantification(quantifier) and the other two as qualifier to unmistakably identify the analytes.

### 2.9. Calibration Curves and Method Validation

Calibration curves were calculated from the peak-area ratio of each analyte quantifier transition to the deuterated IS; the ratio was then plotted on the y-axis against the nominal analyte concentration to generate the standard curves by the method of least squares using a weighed (1/x) linear regression model.

The derivatization procedure for progesterone and 5α-DHP, whose molecules bear two keto groups, led to two cis/trans isomers, which were well resolved in the LC-MS/MS chromatograms; therefore, for these analytes quantification was based on the sum of both peak areas. All calibration curves exhibited R^2^ values >0.990.

The limit of quantitation (LOQ) for each target analyte was evaluated from the corresponding calibration plots as 10 σ/S, where σ and S are the standard deviation and the slope of the regression line. The calculated LOQ values (0.2 pg/700 µL for allopregnanolone, progesterone and pregnanolone sulfate and 0.5 pg/700 µL for the other neurosteroids) were then validated by analyzing blank samples spiked with the analytes at the calculated LOQ levels. The LC–MS/MS method was also validated with respect to precision, accuracy and recovery.

The intra- and inter-day precision were evaluated by calculating the percent relative standard deviation (RSD%) of replicate analyses of three calibrators at different concentration level within the calibration range (low, medium, high); accuracy of the method was evaluated by comparing the levels found in spiked blank samples with the nominal analyte concentration. The intra-assay and inter-assays coefficients were satisfactory, being the related RSD% values lower than 11% and 14%, respectively, for all analytes at all tested concentration levels, which comply with the EMA guidelines [36]. Accuracy values were in the range ± 12%, which are within the accepted limits for this parameter [36]. Recovery for the developed procedure was calculated by comparing the response of post-spiked samples to that of the pre-spiked samples and the obtained values were > 92% for all the analytes.

### 2.10. Statistical Analysis

The Graph-Pad Prism program (GraphPad Software Inc., San Diego, CA, U.S.A.) was used for data analysis of viability and ROS assay (one-way ANOVA and Bonferroni post-hoc test) and graphic presentation. Secifically we use the non-linear regression analysis to generate the curve and IC_50_ value. Data analysis of neurosteroids determination were compared using two-way ANOVA and Holm-Šídák post-hoc test (Sigmaplot 11; Systat Software, San Jose, CA, U.S.A.). We used Shapiro–Wilk test as a test of normality and it passed for the analysis of all data analyzed. All data are presented as the mean ± SEM. The F distribution values are reported for all results. Statistical analysis was performed as indicated in each graphic. The *p*-value <0.05 was considered to be statistically significant.

The authors prepared references using Zotero as bibliography software.

## 3. Results

### 3.1. Viability of BV-2 Cells Exposed to Rotenone

**Figure 1:**
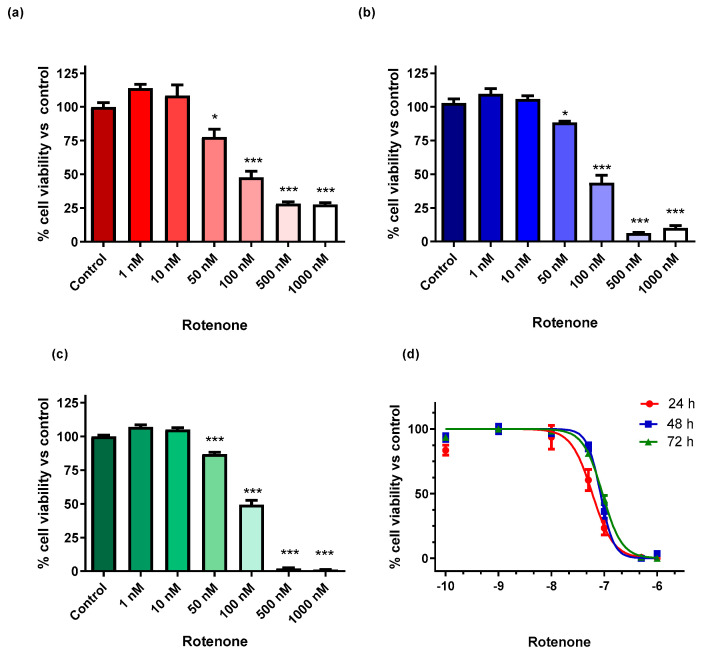
Viability of BV-2 cells exposed to different rotenone concentrations (1–1000 nM) at progressively longer time intervals: (**a**) 24 h, (**b**) 48 h and (**c**) 72 h. (**d**) Rotenone toxicity curves for BV-2 cell viability over time. The data represent the average ± SEM of 3 independent experiments (*n* = 6). * *p* < 0.05 and *** *p* < 0.0001 vs control, according to one-way analysis of variance (ANOVA) followed by Bonferroni post hoc test for multiple comparisons.

BV-2 cell viability was significantly reduced 24 h after exposure at 50, 100, 500 and 1000 nM rotenone different concentrations (F_6, 35_ = 62.73; *p* < 0.05 for rotenone 50 nM, and *p* < 0.001 for rotenone 100, 500 and 1000 nM, respectively, compared to control levels; one-way analysis of variance –ANOVA - followed by Bonferroni correction) (Figure 1a). As expected, these effects of rotenone exposure were confirmed at the 48 h time interval (F_6, 35_ = 210.0; *p* < 0.05 for rotenone 50 nM, and *p* < 0.001 for rotenone 100, 500 and 1000 nM, respectively, compared to control levels) (Figure 1b) and even more pronounced at the 72 h time interval (F_6, 35_ = 865.6; *p* < 0.001 for rotenone 50–1000 nM vs. control levels) (Figure 1c). Rotenone (1–1000 nM) reduced BV-2 cells’ viability with an IC_50_ of 59.5 nM ± 10.9 (mean ± standard error of the mean, SEM) after 24 h (R = 0.9035), increasing to 84.9 nM ± 10.4 after 48 h (R = 0.9688) and 92.6 nM ± 10.3 after 72 h (R = 0.9913) (Figure 1d) to suggest the progressive development of resistance to rotenone toxicity. Intra- and inter-assay variations for BV-2 cell viability were, respectively, 13.8–18.1% and 14.9–20.4%, calculated by considering both treatment conditions in the time interval of 24–72 h.

### 3.2. Determination of ROS

**Figure 2:**
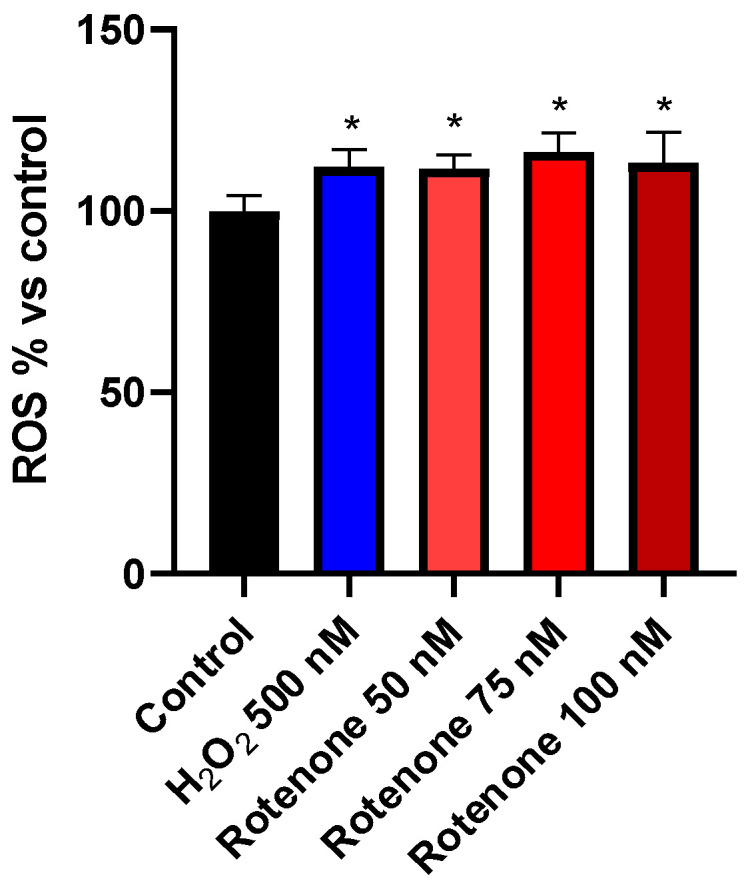
Histograms illustrating ROS production in BV-2 cells treated with increasing concentrations of rotenone (50–100 nM). Each column represents the average ± SEM of the percentage of the fluorescence, normalized with respect to the control, resulting from data of 3 independent experiments (*n* = 6). * *p* < 0.05 vs control, one-way ANOVA followed by Bonferroni post hoc test.

Exposure to increasing doses of rotenone (50–100 nM) determined a significant but dose-independent increase in ROS production (Figure 2), which was similar to that determined with exposure to the strong oxidant H_2_O_2_ (500 nM) (F_4, 25_ = 7.807; *p* < 0.05 vs. control levels for all rotenone concentrations as well as for H_2_O_2_, one-way ANOVA followed by Bonferroni correction). This finding suggested the presence of a ceiling effect for ROS production and the involvement of mechanisms other than oxidation in the progressive reduction of cell viability illustrated by Figure 1. The intra-assay and inter-assay coefficients of variation for cell viability, respectively, were within the range of 4.2–7.5% and 6.6–8.4%, considering all conditions at the 4 h time interval.

### 3.3. Effect of Rotenone Exposure on Neurosteroid Levels in BV-2 Cell Medium

Pregnenolone, pregnenolone sulfate, progesterone, 5α-DHP, pregnanolone, and allopregnanolone were analyzed by liquid chromatography with tandem mass spectrometry (LC-MS/MS) using the culture medium of resting and, respectively, activated BV-2 cells after exposure to the threshold dose of rotenone (75 nM) for 24, 48 and 72 h. The dose of rotenone was chosen to induce a microglial activation below the IC_50_ to get a sufficient number of alive cells to evaluate the neurosteroid levels.

**Figure 3:**
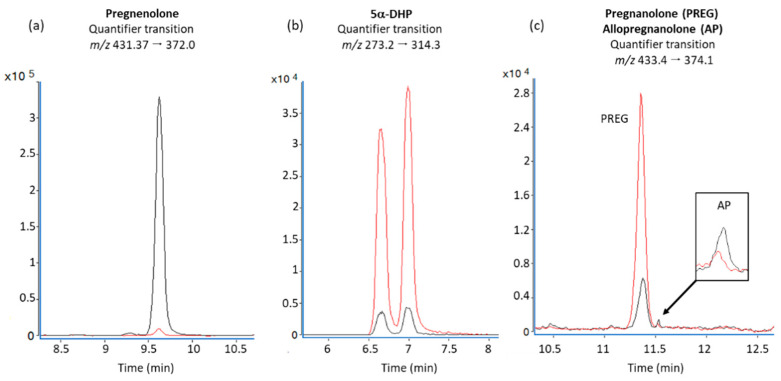
MRM quantifier transition for (**a**) pregnenolone, (**b**) 5α-DHP, (**c**) pregnanolone and allopregnanolone as found in the medium of BV-2 cells at the 72 h time interval (black traces: resting cells; red traces: cells activated by rotenone).

Figure 3 shows representative peaks of the multiple reaction monitoring (MRM) quantifier transition for pregnenolone, 5α-DHP, pregnanolone and allopregnanolone in resting microglia (black traces) and activated microglia (red traces) at 72 h post-exposure.

**Figure 4:**
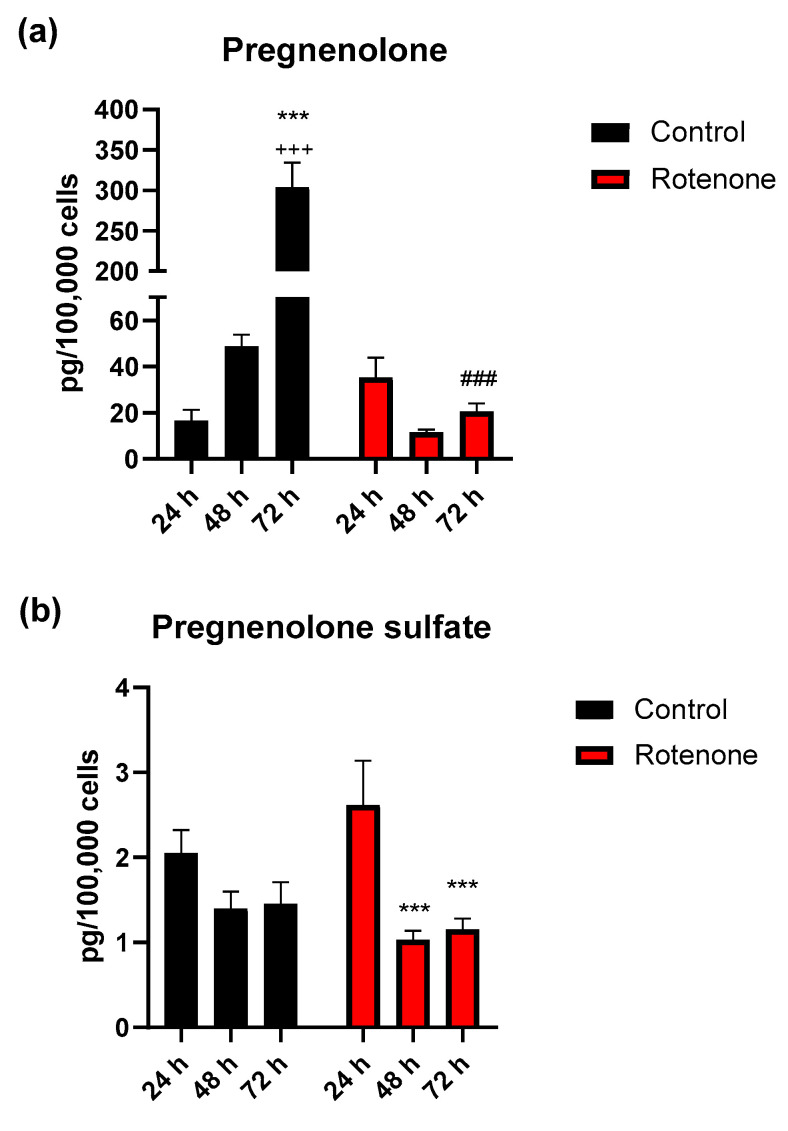
Pregnenolone (**a**) and pregnenolone sulfate (**b**) concentrations in BV-2 cells’ medium determined by LC-MS/MS in microglia, in resting condition (Control) or after activation by exposure to rotenone at different time intervals (24, 48 and 72 h). Data represent the average of 2 independent experiments (*n* = 5) and are expressed in pg/100,000 cells (mean values ± SEM). Statistical analysis was by two-way ANOVA and Holm-Šídák post hoc test. Pregnenolone: *** *p* < 0.001, vs 24 h control; ^+++^ *p* < 0.001, vs 48 h control; ^###^ *p* < 0.001, vs same time interval of control. Pregnenolone sulfate: *** *p* < 0.001, vs 24 h rotenone.

According to statistical analysis of pregnenolone levels, we found a main effect for both time (F_2, 23_ = 81.02, *p* < 0.001, two-way ANOVA) and treatment (F_1, 23_ = 99.87, *p* < 0.001), as well as a significant interaction of these factors (F_2, 23_ = 87.80, *p* < 0.001). Pregnenolone levels in the culture medium of resting BV-2 cells, expressed as pg/100,000 cells, were 16.5 ± 4.78 (mean value ± SEM, 2 independent experiments *n* = 5). After 48 h, its concentration increased up to 295% without reaching a statistically significant level (*p* = 0.09, 48 h vs 24 h). At 72 h, pregnenolone increased up to 1841% and 624%, respectively, vs 24 h or 48 h (*p* < 0.001 for both time intervals, Holm-Šídák post hoc test) (Figure 4a). Exposure of BV-2 cells to rotenone did not significantly change the concentration of pregnenolone in the culture medium over time. By comparing the control group to rotenone-treated BV-2 cells, a significant difference in pregnenolone level was determined for treatment at the 72 h time interval only (*p* < 0.001).

In the case of pregnenolone sulfate, two-way ANOVA revealed a statistically significant difference only for time (F_2, 23_ = 11.36, *p* < 0.001). After 24 h, pregnenolone sulfate levels in the culture medium of resting BV-2 cells were 2.0 ± 0.27 pg/100,000 cells (2 independent experiments, *n* = 5) and did not change after 48 or 72 h. The concentration of pregnenolone sulfate in the BV-2 cell medium 24 h after exposure to rotenone was 2.6 ± 0.53 pg/100,000 cells and decreased significantly to 32% at 48 h (*p* < 0.001), then maintained at 58% of basal values at 72 h (*p* < 0.001) (Figure 4b).

**Figure 5:**
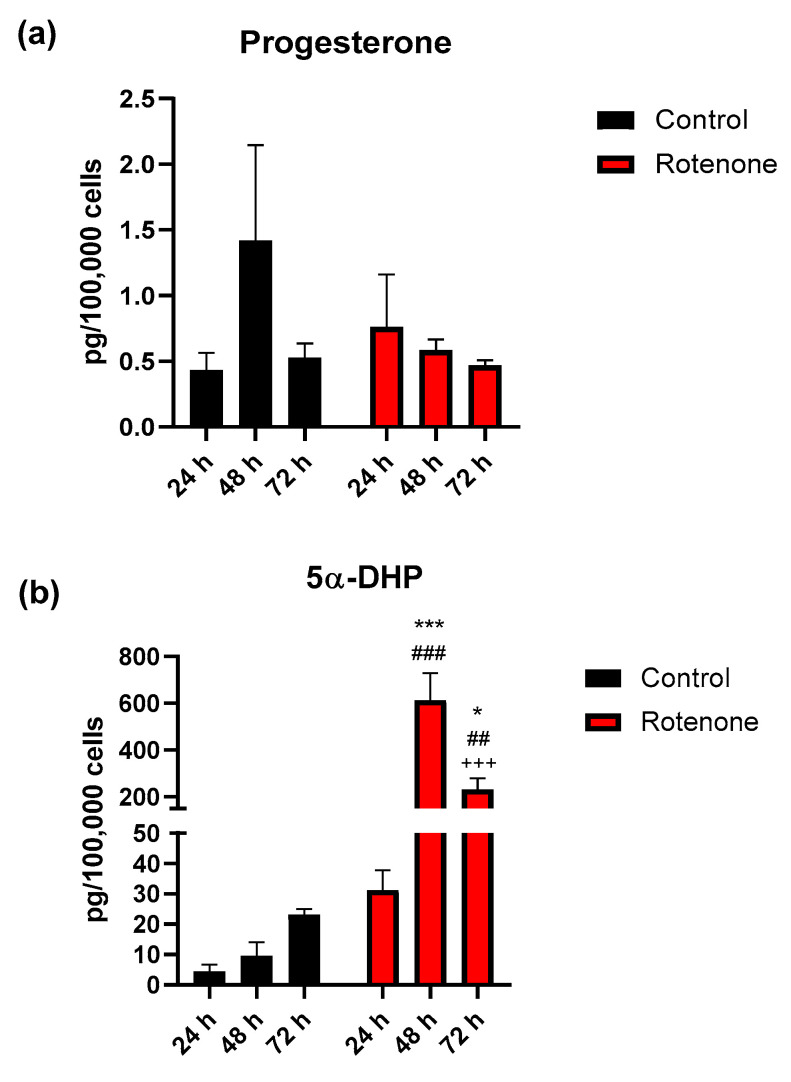
Progesterone (**a**) and 5α-dihydroprogesterone (5α-DHP) (**b**) levels in BV-2 cells’ medium determined by LC-MS/MS in microglia, in resting condition (Control) or after activation by exposure to rotenone at different time intervals (24, 48 and 72 h). Data represent the average of 2 independent experiments (*n* = 5) and are expressed in pg/100,000 cells (mean values ± SEM). Statistical analysis was by two-way ANOVA and Holm-Šídák post-hoc test. 5α-DHP: *** *p* < 0.001, * *p* < 0.05 vs 24 h rotenone; ^+++^ *p* < 0.001, vs 48 h rotenone; ^###^ *p* < 0.001, ^##^ *p* < 0.01, vs same time interval of control.

Progesterone at 24 h was 0.4 ± 0.13 pg/100,000 cells in the culture medium of resting BV-2 cells (2 independent experiments, *n* = 5), and this concentration increased up to 350% after 48 h without reaching a statistically significant level. At the 72 h time interval, progesterone levels were similar to initial values. The concentration of progesterone in the medium of the BV-2 cells exposed to rotenone after 24 h was 0.8 ± 0.40 pg/100,000 cells and did not change in the following time intervals (Figure 5a). Overall, progesterone levels were not affected by time or treatment.

At variance with results for progesterone, 5α-DHP levels were highly significantly affected by time (F_2, 22_ = 18.42, *p* < 0.001) and treatment (F_1, 22_ = 47.68, *p* < 0.001), with a significant interaction (F_2, 22_ = 18.31, *p* < 0.001). Specifically, after 24 h 5α-DHP in the BV-2 cell culture medium was at a concentration of 4.5 ± 2.25 pg/100,000 cells (2 independent experiments *n* = 5) and did not undergo significant changes after 48 or 72 h. The concentration of 5α-DHP in the medium of BV-2 cells exposed to rotenone was 31.2 ± 6.51 pg/100,000 cells after 24 h, and significantly increased up to 1966% after 48 h (*p* < 0.001, 48 h vs 24 h), then maintained to 742% after 72 h (*p* < 0.05, 72 vs 24 h) (Figure 5b). However, 5α-DHP levels at 72 h were significantly decreased in comparison to the 48 h time interval (*p* < 0.001). By comparing the control group with BV-2 cells exposed to rotenone, a significant difference in 5α-DHP levels was determined for treatment at 48 h (*p* < 0.001, 48 h rotenone vs 48 h control) and 72 h (*p* < 0.01).

**Figure 6:**
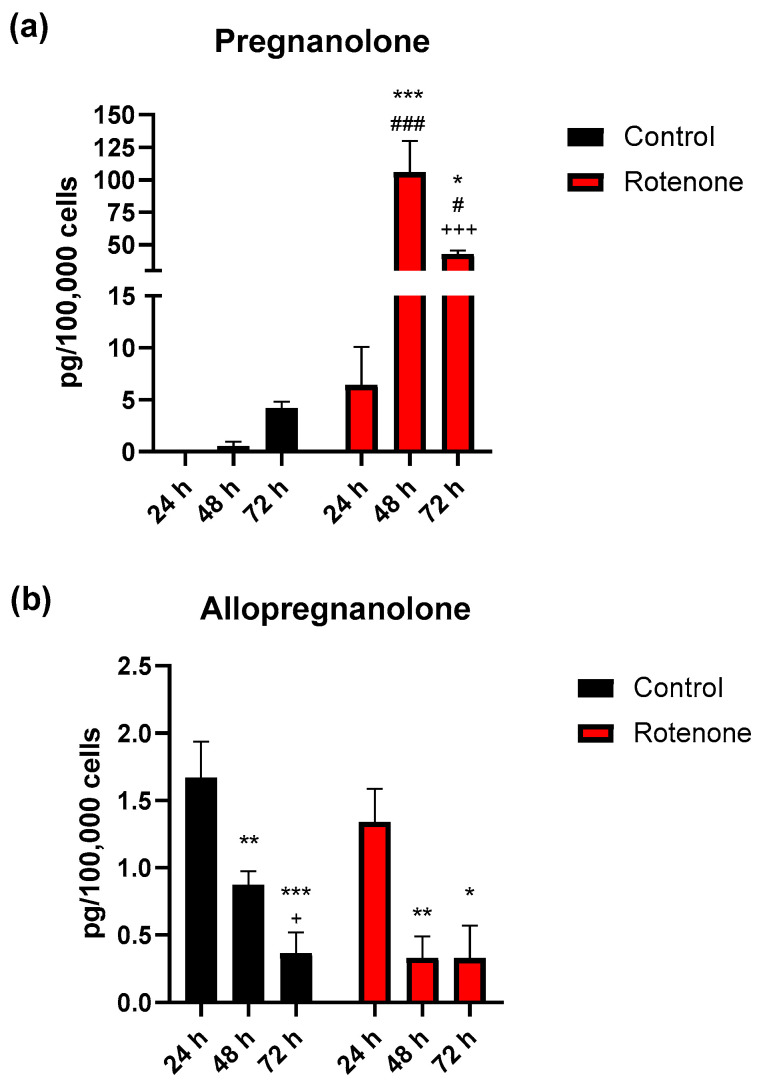
Pregnanolone (**a**) and allopregnanolone (**b**) concentration in BV-2 cells medium determined by LC-MS/MS in microglia, in resting condition (Control) or after activation by exposure to rotenone at different time intervals (24, 48 and 72 h). The data represent the average of 2 independent experiments (*n* = 5) and are expressed in pg/100,000 cells (mean values ± SEM). The significance of the differences was determined by two-way ANOVA followed by Holm-Šídák post-hoc test. Pregnanolone: *** *p* < 0.001, * *p* < 0.05, vs 24 h rotenone; ^+++^ *p* < 0.001, vs 48 h rotenone; ^###^ *p* < 0.001, ^#^ *p* < 0.05, vs same time interval of control. Allopregnanolone: *** *p* < 0.001, ** *p* < 0.01, vs 24 h control or rotenone; * *p* < 0.05, vs 24 h rotenone; ^+^ *p* < 0.05, vs 48 h control.

Also, pregnanolone levels were highly significantly affected by time (F_2, 22_ = 13.70, *p* < 0.001) and treatment (F_1, 22_ = 39.73, *p* < 0.001), with a significant interaction (F_2, 22_ = 14.03, *p* < 0.001). Pregnanolone levels measured in the control BV-2 cell culture medium did not undergo significant changes after 24, 48 or 72 h. Instead, pregnanolone levels in the medium of BV-2 cells exposed to rotenone were 6.4 ± 3.66 pg/100,000 cells; then, they increased significantly up to 1653% at 48 h (*p* < 0.001, 48 h vs 24 h and, respectively, 72 h) and did not recover, although reduced to 669% of basal levels at 72 h (*p* < 0.001 vs 48 h of the same group, *p* < 0.05 vs 24 h) (Figure 6a). Additionally, a significant difference in pregnanolone levels was found at both 48 h (*p* < 0.001) and 72 h (*p* < 0.05) with respect to the same time interval of the control group.

Statistical analysis of allopregnanolone levels revealed a significant effect for time (F_2, 24_ = 22.08, *p* < 0.001) but not for treatment. Allopregnanolone levels were 1.7 ± 0.27 pg/100,000 cells after 24 h in the control BV-2 cell culture medium (2 independent experiments, *n* = 5), and significantly decreased to 52% at 48 h and to 23% at 72 h of 24 h values (*p* < 0.001, 24 h vs 72 h; *p* < 0.01, 24 h vs 48 h). In the culture medium of BV-2 cells exposed to rotenone, the concentration of allopregnanolone was 1.3 ± 0.25 after 24 h and significantly decreased to 23% at both 48 h (*p* < 0.01) and 72 h (*p* < 0.05) time intervals (Figure 6b).

## 4. Discussion

In the present study, we provide the first evidence for the synthesis of different neurosteroids in cultivated microglia. Specifically, pregnenolone, pregnenolone sulfate, progesterone, 5α-DHP, allopregnanolone, and pregnanolone significantly changed over time in the culture medium of BV-2 cells, thus demonstrating that microglia are capable of synthesizing neurosteroids up to final products such as allopregnanolone and pregnanolone, two potent modulators of the γ-aminobutyric acid (GABA) type A (GABA_A_) receptor.

Until now, different authors reported the synthesis of neurosteroids in astrocytes, oligodendrocytes and neurons [24], and these molecules have been measured in the cerebral tissue [37] or cerebrospinal fluid [38]. Only scanty reports proposed a similar possibility also for the microglia, but with incomplete evidence on a fully expressed metabolic pathway leading to final and active neurosteroid products [28,39].

We also tested the synthesis of neurosteroids in the presence of a toxicant used to mimic the PD in vitro. Interestingly, in the presence of enhanced oxidation and limited cell death induced by rotenone, levels of 5α-DHP and pregnanolone markedly increased, instead pregnenolone sulfate and allopregnanolone were significantly reduced, whereas pregnenolone and progesterone were precluded to change their levels over time as instead observed in the control medium of the BV-2 microglial cells. These findings suggest that the metabolic pathway of the microglia is dynamically regulated so as to readily respond to injuring stimuli associated with neurodegeneration. It remains to be examined if the observed changes could result in the protection or not of the microglia exposed to rotenone, and the possible consequences for neuronal death. Rotenone exerts a pro-inflammatory effect on microglial cells through inhibition of the mitochondrial respiratory chain, causing the ROS increase and oxidative stress. We confirmed rotenone toxicity by treating BV-2 cells with increasing concentrations of this toxicant (50, 75, 100 nM). Indeed, it is well documented that excessive production of ROS from activated microglia plays an important role in the process of neurodegenerative diseases [40]. Therefore, we aimed at confirming that rotenone in the range of concentrations used for BV-2 treatments was able to increase ROS content, which is involved in the initiation, progression, and resolution of the inflammatory response. Moreover, in response to neuroinflammation and neurodegenerative diseases, such as PD [41], the TSPO expression significantly increases in microglia and astrocytes so that TSPO itself has been reported as a marker for microglial activation [42,43]. Among the various functions such as porphyrin transport and heme synthesis, apoptosis, cell proliferation, anion transport, regulation of mitochondrial functions and immunomodulation, TSPO is implicated in the regulation of cholesterol transport and in the synthesis of steroid hormones [44,45,46].

Allopregnanolone was found to be consistently reduced in different neurodegenerative diseases, including PD [47], AD [48], and MS [49]. The results obtained in our experiments are in agreement with such reports and, in view of the possible meaning of the investigated model, are relevant for the PD. However, our results highlight a new phenomenon not previously reported: the reduction in some neurosteroids (i.e., allopregnanolone and pregnenolone sulfate) is compensated by an even more marked increase in other neurosteroids (5α-DHP and pregnanolone). This finding suggests that the neurosteroid pathway could be hyperfunctional instead of defective. However, a possible compensation for the reduction in some neurosteroid production is supposed to occur when the involved species have similar effects. This is indeed the case for allopregnanolone, since pregnanolone is known to interact with the same binding site for allopregnanolone in the GABA_A_ receptor, resulting in superimposable effects [50]. Since pregnanolone potentiates the inhibitory currents generated by the GABA_A_ receptor, which are instead counteracted by pregnenolone sulfate, the overall balance could be favorable for the GABAergic tone.

The neuroprotective effect of neurosteroids can be explained by different mechanisms of action. Progesterone acts via genomic mechanisms after binding to the classical intracellular receptors (PR) or has rapid membrane actions via specific membrane receptors (mPR) or membrane binding sites (PGRMC1). Some effects of progesterone may be mediated by its metabolites, for example 5α-DHP binds to the classical receptors PR and has relatively high binding affinity for mPRα [51]. Thus, it is interesting to observe that 5α-DHP levels were highly increased by rotenone.

Allopregnanolone and pregnanolone equipotently and positively modulate the action of GABA at GABA_A_ synaptic and extrasynaptic receptors. At nanomolar doses, these stereoisomers increase the effects of GABA on GABA_A_ receptor-mediated chloride ion influx 7 to 10 times, thus markedly improving the inhibitory impact of GABA on neuronal firing [21,52]. In contrast, sulfated, allopregnanolone, and pregnanolone potently antagonize N-methyl-D-aspartate (NMDA) receptors [52]. However, allopregnanolone can activate gene transcription via PR after being converted back to 5α-DHP. Some neuroprotective effects of allopregnanolone may be mediated by the pregnane X receptor (PXR) or by the membrane progesterone receptor mPRδ [51]. On the other hand, allopregnanolone binds to GABA_A_ receptors in microglia as well as astrocytes mediating anti-inflammatory effects through nuclear factor-κB (NF-kB) inhibition [53,54,55].

In addition to these pharmacological effects, neurosteroids can suppress inflammation, reduce apoptosis, and promote neurogenesis and myelination [40]. Allopregnanolone can also act on the Purkinje cell for the survival of this neuron by suppressing the expression of caspase-3, a crucial mediator of apoptosis [56]. Moreover, the protective effects of allopregnanolone in immune cells and the brain involve blocking of protein-protein interactions that initiate Toll-like receptor 4 (TLR4)-dependent signaling. Inhibition of pro-inflammatory TLR4 activation represents a new mechanism of allopregnanolone action in the periphery and the brain [57].

Over the past decade, the important concept has emerged that microglia, like macrophages in various tissues, can be present in different phenotypes and perform different effector functions. This principle generates the theory that activated microglia can have pro-inflammatory or anti-inflammatory and repairing functions. It is essential to underline that microglia in vivo present with a homeostatic or a disease-associated/neurodegenerative phenotype [12], whereas in vitro differences in microglia phenotypes are classically indicated as M1 and M2 polarization when cells could be made reactive by damaging stimuli. Neuroinflammation and microglia activation are neuropathological distinctive marks in Parkinson’s disease. However, their role in the progression of the disease has not yet been clarified, therefore, it is a challenge to discover new therapies for the PD prevention and treatment.

There is also a metabolic issue. In in vitro condition, the M1/pro-inflammatory phenotype is associated with the inhibition of the respiratory chain and the increase in glycolysis, which results in a faster ATP production, thus corresponding to the metabolic needs of the M1 phenotype itself. In this situation, the glycolytic and pentose phosphate pathways are enhanced, and oxidative phosphorylation is inhibited [58]. Overall, microglia cells can manage the metabolic changes induced by rotenone more efficiently than astrocytes. One possible explanation is that while activated microglial cells switch to glycolysis, astrocytes exposed to pro-inflammatory stimuli increase the activity of tricarboxylic acid cycle. 

The synthesis of neurosteroids in microglial BV-2 cell line has been supported by our findings. The original result of our research suggests that the increase in some neurosteroids in activated microglia could protect the neuron from inflammatory insult. One could speculate that once activated, microglia initially release inflammatory cytokines and, subsequently, anti-inflammatory neurosteroids. The fact that the TSPO is more expressed in the activated microglia suggests a greater translocation of cholesterol from the outside to the inside of the mitochondrial membrane, where the synthesis of neurosteroids begins thanks to the presence of cytochrome P450. In fact, a characteristic of activated microglia, following an insult to the CNS, is the larger expression of TSPO. The determination of neurosteroids such as pregnenolone, pregnenolone sulfate, progesterone, 5α-DHP, pregnanolone, and allopregnanolone in resting and activated BV-2 cell line, as illustrated by our results, represents information on the possible in vivo role of microglia in modifying the microenvironment in which neuroinflammation modulates neuronal survival.
